# Gintonin-Enriched *Panax ginseng* Extract Fraction Sensitizes Renal Carcinoma Cells to TRAIL-Induced Apoptosis through DR4/5 Upregulation

**DOI:** 10.3390/cimb46100646

**Published:** 2024-09-27

**Authors:** Seongwoo Hong, Rami Lee, Gyun Seok Park, Sumin Han, Juhyun Shin, Yoon-Mi Lee, Seung-Yeol Nah, Jae-Wook Oh

**Affiliations:** 1 Department of Stem Cell and Regenerative Biotechnology, Konkuk University, Seoul 05029, Republic of Korea; hsw2332@naver.com (S.H.); hsm534@naver.com (S.H.); junejhs@gmail.com (J.S.); dldbsal1414@naver.com (Y.-M.L.); 2 Ginsentology Research Laboratory, Department of Physiology, College of Veterinary Medicine, Konkuk University, Seoul 05029, Republic of Korea; rmlee12@konkuk.ac.kr (R.L.); synah@konkuk.ac.kr (S.-Y.N.); 3 Department of Bio-Resources and Food Science, Konkuk University, 120 Neungdong-ro, Gwangjn-gu, Seoul 05029, Republic of Korea; bhs2945@hanmail.net

**Keywords:** tumor necrosis factor-related apoptosis-inducing ligand, gintonin-enriched fraction, human renal cell carcinoma, apoptosis, mu-2-related death-inducing gene

## Abstract

Tumor necrosis factor-related apoptosis-inducing ligand (TRAIL) is a promising chemotherapeutic agent because of its selective apoptotic action on cancer cells. However, resistance to TRAIL-induced apoptosis remains a challenge in many cancers. The gintonin-enriched *Panax ginseng* extract fraction (GEF) has diverse pharmacological benefits. We explored the combined efficacy of GEF and TRAIL in inducing apoptosis in human renal cell carcinoma (RCC) cells. The effect of GEF treatment on the viability, clonogenic potential, wound healing, and TRAIL-induced apoptotic signaling of RCC cells was studied in vitro. Our investigation revealed that GEF pre-treatment sensitized RCC cells to TRAIL-induced apoptosis, as evidenced by DNA fragmentation and cell proliferation, colony formation, and migration inhibition. This sensitization was linked to the upregulation of death receptors 4 and 5 and alterations in apoptotic protein expression, notably, the decreased expression of the Mu-2-related death-inducing gene, a novel anti-apoptotic protein. Our findings underscore the necessity of caspase activation for GEF/TRAIL-induced apoptosis using the pan-caspase inhibitor Z-VAD-FMK. This study demonstrates that GEF sensitizes human RCC cells to TRAIL-induced apoptosis by upregulating DR4/5 and modulating apoptotic protein expression. These findings suggest a promising strategy for overcoming TRAIL resistance in cancer therapy and highlight the potential of GEF as a valuable adjunct to TRAIL-based treatments.

## 1. Introduction

Renal cell carcinoma (RCC) is the most common kidney cancer and a global health concern [1], with over 400,000 new cases reported in 2020 [2]. Although surgery is the standard treatment for localized RCC, the recurrence rate is high [3]. Advanced RCC treatment options, such as immunotherapy and targeted therapies, are promising but have limited efficacy [4,5,6]. Therefore, safer and more effective treatments for metastatic RCC are urgently needed.

Tumor necrosis factor-related apoptosis-inducing ligand (TRAIL), a key member of the tumor necrosis factor (TNF) superfamily, selectively induces apoptosis in cancer cells [7,8] by binding to death receptors 4 and 5 (DR4/5) [9], triggering intracellular signaling and leading to programmed cell death [10]. However, cancer cells often resist TRAIL-induced apoptosis because of altered receptor expression and apoptotic protein dysregulation [11]. Combining TRAIL-based therapies with agents that sensitize cancer cells to apoptosis holds promise in overcoming drug resistance and advancing therapeutic strategies.

Research has highlighted the potential therapeutic effects of various natural products against RCC. Compounds such as kahweol acetate, honokiol, englerin A, and epigallocatechin-3-gallate have shown diverse anti-cancer properties in RCC [12,13]. Furthermore, recent studies have indicated that 17β-Hydroxywithanolides and their analogs can sensitize RCC cells to apoptosis induced by TRAIL, enhancing their susceptibility to this form of programmed cell death [14,15,16].

Ginseng, a revered herb in Asia, has potential health benefits due to its adaptogenic properties [17]. Recent studies have identified gintonin, a novel glycolipoprotein distinct from traditional ginsenosides, which activates G protein-coupled receptors, particularly LPA receptors [18,19]. A gintonin-enriched *Panax ginseng* extract fraction (GEF) was prepared to harness the potency of gintonin through ethanol extraction and water fractionation [20]. GEF and gintonin exhibit diverse pharmacological effects, including neuronal protection, anti-inflammatory action, and the suppression of cancer cell metastasis [21,22,23,24].

Mu-2-related death-inducing gene (MUDENG, MuD), a novel gene structurally similar to the Mu-2 adaptin subunit, is involved in intracellular trafficking mechanisms [25,26] and apoptotic signaling pathways [26,27,28,29]. It plays a role in TRAIL-induced apoptosis by interacting with key molecules, such as caspase-3, BH3 interacting-domain death agonist (Bid), and B-cell lymphoma (Bcl)-2 [29].

In this study, we explored the effects of GEF on TRAIL-induced apoptosis in human RCC cells. Our findings reveal that GEF augmented DR4/5 expression and potentiated caspase-dependent TRAIL-induced apoptosis in these cells.

## 2. Materials and Methods

### 2.1. GEF Preparation

GEF was extracted and lyophilized from *Panax ginseng* as described previously [20]. The dried GEF powder was stored at −80 °C and dissolved in dimethyl sulfoxide (DMSO) prior to cell treatment.

### 2.2. LC-MS/MS-Based Quantification of Lysophospholipids (LPLs) and Phospholipids (PLs) in GEF

Lysophospholipids (LPLs) and phospholipids (PLs) were quantified following the method of Cho et al. [30]. A gintonin-enriched fraction (100 mg) was dissolved in 10 mL of water, vortex-mixed, and 1 mL of the middle layer was diluted with methanol, filtered, and injected (2 µL) for LC-MS/MS analysis. Stock solutions of LPLs and PLs were prepared in HPLC-grade methanol and stored at 4 °C. Quantification was performed using an Agilent 1100 HPLC system coupled with an API 2000 LC-MS/MS system in multiple reaction monitoring (MRM) mode. Analytes were separated using XBridge C18 and Hydrophilic Interaction columns (Waters Corporation) with a mobile phase of acetonitrile, formic acid, and ammonium formate. Electrospray ionization was conducted in positive mode for LPC and PC, and negative mode for LPA, LPE, LPI, PA, and PI. The optimized MS/MS conditions included ion spray voltages of −3.5 to −4.5 kV (negative mode) and 5.5 kV (positive mode), a capillary temperature of 400–450 °C, and specific gas flows for nebulizing, auxiliary, and curtain gases. Quantification was based on the MRM of precursor and product ions using an internal standard method with peak area ratios.

### 2.3. Cell Culture

Human RCC cells (786-O, A498) from ATCC (Manassas, VA, USA) were cultured in RPMI 1640 and DMEM supplemented with 10% FBS, 10,000 U/mL penicillin, and 10,000 μg/mL streptomycin (Welgene, Gyeongsan, Republic of Korea) at 37 °C with 5% CO_2_.

### 2.4. Cell Viability Assay

The cells (4.0 × 10^3^ cells/well) were seeded in 96-well plates and cultured overnight. After serum deprivation, the cells were treated with various GEF concentrations or DMSO (control) for 12 h. Subsequently, they were exposed to different TRAIL/Apo2L concentrations (PeproTech, Cranbury, NJ, USA) for another 12 h. Cell viability was assessed with 4-[3-(4-iodophenyl)-2-(4-nitrophenyl)-2H-5-tetrazolio]-1,3-benzene disulfonate (WST-1) assay using the EZ-cytox kit (Dogen, Seoul, Republic of Korea); the absorbance was measured at 450 nm with a reference wavelength of 600 nm using a SpectraMax M2e microplate reader (Molecular Devices, San Diego, CA, USA).

### 2.5. Colony Formation Assay

The cells (1.5 × 10^5^ cells/well) were seeded in 6-well plates overnight, pre-treated with GEF or DMSO for 12 h in serum-free medium, and exposed to TRAIL for another 12 h. The cells were harvested, reseeded (6 × 10^2^ cells/well) in 6-well plates, and cultured for 10 days. The colonies were fixed with 100% methanol, stained with 0.5% crystal violet, and counted using ImageJ software (Version 1.8.0, National Institute of Health, Bethesda, MD, USA).

### 2.6. Wound Healing Assay

The cells (2.5 × 10^5^ cells/well) were seeded in 6-well plates and allowed to adhere overnight. A scratch was made in the center of each well, followed by washing with a serum-free medium. The cells were then pre-treated with GEF or DMSO for 12 h before treatment with or without TRAIL for another 12 h. Wound images were captured using a light microscope (OLYMPUS CKX41, Tokyo, Japan), and the wound size after scratching was quantified using ImageJ software. Then, the wound size was quantified again after treatment for 24 h to indicate how much wound closure (%) was achieved through comparison of the two-wound size.

### 2.7. Apoptosis Assay Using FACs

The cells (1.5 × 10^5^) seeded in 6-well plates were pre-treated with 20 µM Z-VAD-FMK (R&D Systems, Minneapolis, MN, USA) for 1 h before exposure to GEF or DMSO for 12 h. Subsequently, the cells were treated with or without TRAIL for another 12 h. Apoptosis was assessed using the fluorescein isothiocyanate (FITC)—Annexin V Apoptosis Detection Kit (BD Biosciences, San Diego, CA, USA), and flow cytometric analysis was conducted using NovoCyte 1000 with data visualization using NovoExpress software (version 1.2.5, ACEA Biosciences, San Diego, CA, USA). Data analysis was performed according to the cell population in the quadrant. The quadrants were divided into Q3-1 (Annexin V−/PI+, necrotic cells), Q3-2 (Annexin V+/PI+, late apoptotic cells), Q3-3 (Annexin V−/PI−, healthy cells), and Q3-4 (Annexin V+/PI−, early apoptotic cells), respectively. The apoptotic cell population was measured by combining the cell populations of Q3-2 (late apoptotic cells) and Q3-4 (early apoptotic cells).

### 2.8. Western Blot Analysis

The cells (2.5 × 10^5^ cells/well in 60-mm dishes) were seeded and allowed to adhere overnight before pre-treating with GEF or DMSO for 12 h in a serum-free medium. Subsequently, the cells were treated with TRAIL for 12 h. After treatment, cell lysates (30 μg) were prepared and subjected to 10–12% SDS–PAGE. Polyvinylidene difluoride (PVDF) membranes (Bio-Rad, Hercules, CA, USA) were used for protein transfer and blocked with 5% skim milk before incubation with primary antibodies against DR4 (cat. no. sc-8411), Bax (cat. no. sc-7480), Bcl-2 (cat. no. sc-7382), β-actin (cat. no. sc-47778) (Santa Cruz Biotechnology, Dallas, TX, USA), DR5 (cat. no. ab8416) (Abcam, Cambridge, MA, USA), PARP (cat. no. 9542S), caspase-3 (cat. no. 9662S), Bid (cat. no. 2002S) (Cell Signaling Technology, Danvers, MA, USA), and MuD proteins [31]. After washing, the membranes were incubated with horseradish peroxidase-conjugated secondary antibodies (Jackson ImmunoResearch, West Grove, PA, USA), and the proteins were visualized using an enhanced chemiluminescence kit (Bio-Rad).

### 2.9. TUNEL Staining

The cells (4.0 × 10^3^ cells/well) were seeded in 96-well plates and cultured overnight. After incubation with GEF or DMSO for 12 h, the cells were treated with or without TRAIL for another 12 h. After fixation with 4% paraformaldehyde and permeabilization with PBST, the TUNEL assay was performed according to the manufacturer’s instructions (Promega, Madison, WI, USA). The cells were then stained with 4′,6-diamidino-2-phenylindole, visualized using an ECLIPSE Ts2R inverted microscope (Nikon, Melville, NY, USA), and imaged using NIS-Elements BR software (Ver 4.00, Nikon).

### 2.10. Cell Surface Receptor Staining for FACS Analysis

The cells (1.5 × 10^5^ cells/well) were seeded in 6-well plates and incubated overnight, pre-treated with GEF or DMSO in serum-free medium for 12 h, and then treated with or without TRAIL for another 12 h. After harvesting and washing with DPBS, the cells were incubated with antibodies against DR4 and DR5 for 1 h on ice, incubated with FITC-conjugated secondary antibodies, washed, and analyzed via flow cytometry using a NovoCyte 1000. The mean fluorescence intensity (MFI) was calculated by analyzing the mean X value of the fluorescence intensity. The data were analyzed using the NovoExpress software (version 1.2.5).

### 2.11. Real-Time Quantitative PCR

The total RNA was extracted using TRIzol reagent (Sigma-Aldrich, St. Louis, MO, USA), and the cDNA was synthesized using the AccuPower^®^ RT Premix (Bioneer, Daejeon, Republic of Korea). SYBR qPCR Mix (CellSafe, Yongin, Republic of Korea) and primers for DR4, DR5, and GAPDH were used: DR4 (forward) 5′-TGTGACTTTGGTTGTTCCGTTGC-3′ and (reverse) 5′-ACCTGAGCCGATGCAACAACAG-3′; DR5 (forward) 5′-AGACCCTTGTGCTCGTTGTC-3′ and (reverse) 5′-TTGTTGGGTGATCAGAGCAG-3′; GAPDH (forward) 5′-CCCTCAACGACCACTTTGTC-3′ and (reverse) 5′-CCACCACCCTGTTGCTGTA-3′. Real-time PCR was performed on a CFX96 Real-Time System (Bio-Rad).

### 2.12. Co-Immunoprecipitation (Co-IP)

The cell lysates were co-immunoprecipitated with antibodies against DR4 (cat. no. sc-8411) and DR5 (cat. no. sc-166624) (Santa Cruz Biotechnology) using the Dynabeads™ Protein G Immunoprecipitation Kit (Thermo Fisher Scientific, Waltham, MA, USA). The equivalent amounts of antibodies were loaded onto the beads for all experimental conditions. The input represents the sample on 10% of the volume used for co-IP. The immunoprecipitated proteins were separated by SDS–PAGE, transferred to PVDF membranes, and probed with antibodies against FADD (cat. no. sc-271748), caspase-8 (cat. no. sc-81656) (Santa Cruz Biotechnology), DR4, and DR5. Immunoreactive bands were visualized using an enhanced chemiluminescence kit (Bio-Rad).

### 2.13. Statistical Analysis

Each experiment was performed at least three times independently, and the results are presented as the mean ± SD. Statistical significance was assessed using Student’s *t*-test, with *p* < 0.05, *p* < 0.01, or *p* < 0.001 considered significant.

## 3. Results

### 3.1. Quantitation of LPLs and PLs in GEF Using LC-MS/MS

Lipid analysis revealed that GEF contained approximately 11% free fatty acids (FFAs), with linoleic acid (C18:2) being the most abundant, followed by palmitic acid (C16:0) and oleic acid (C18:1). GEF also had a significant amount of LPA C18:2, making up 0.19% of the sample, along with smaller amounts of other lysolipids (LPLs) like LPC, LPE, and LPI, resulting in a total LPL content of 0.52%. Phospholipids (PLs) make up about 1.84% of GEF, primarily composed of phosphatidic acids (PAs), including 1.17% PA 16:0-18:2. Notably, phosphatidylethanolamine (PE), phosphatidylglycerol (PG), and phosphatidylserine (PS) were not detected. Overall, GEF’s total lipid content was approximately 13.7%, with linoleic acid, LPA C18:2, and PA 16:0-18:2 being the major components (Table 1).

### 3.2. GEF Enhances TRAIL-Induced Cell Death and Inhibits Proliferation in Human RCC Cells

**Figure 1:**
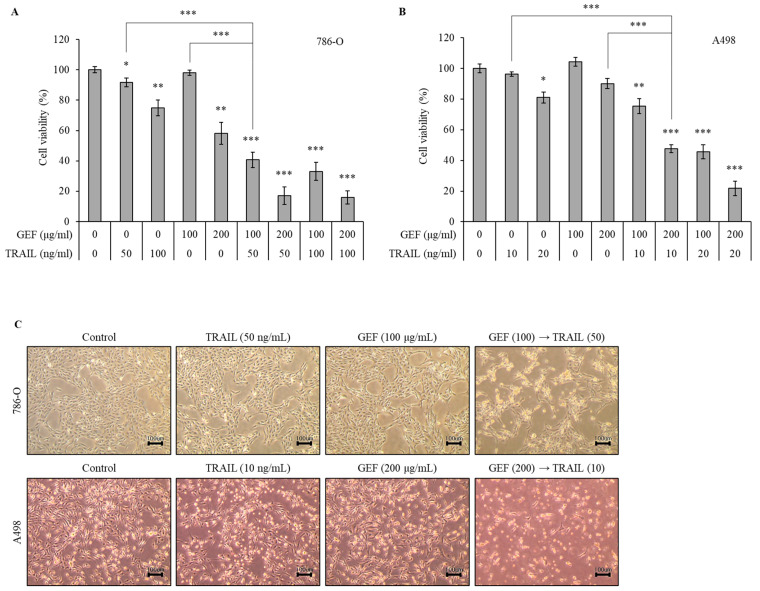
Gintonin-enriched *Panax ginseng* extract fraction (GEF) enhances the sensitivity of human renal cell carcinoma (RCC) cells to tumor necrosis factor-related apoptosis-inducing ligand (TRAIL). (**A**,**B**) 786-O and A498 cells were pre-treated with varying GEF concentrations or DMSO for 12 h, followed by TRAIL treatment with or without GEF for another 12 h. The cell viability was assessed using the WST-1 assay. (**C**) Morphological changes occurred in the cells after 12 h of pre-treatment with GEF followed by TRAIL treatment (40× magnification). The data are represented as the mean ± standard deviation (SD) of three independent experiments, with statistical significance denoted as * *p* < 0.05, ** *p* < 0.01, and *** *p* < 0.001 compared to the untreated control group.

We initially evaluated the effects of GEF and TRAIL, respectively, on RCC cell viability using the WST-1 assay (Supplementary Figures S1 and S2). As shown in Supplementary Figure S1, 786-O was resistant to TRAIL relative to A498 when treated with TRAIL for 24 h. The treatment of 786-O cells with 100 and 200 μg/mL GEF reduced the cell viability dose-dependently (Figure 1A). Pre-treatment with 100 μg/mL GEF followed by a 50 or 100 ng/mL TRAIL treatment significantly reduced the cell viability compared to GEF pre-treatment alone (Figure 1A). Notably, the combination of 100 μg/mL GEF and 50 ng/mL TRAIL synergistically affected the cell viability, suggesting potential cooperation between the two treatments (Figure 1A). This concentration combination was selected for subsequent experiments.

A498 cells were more sensitive to TRAIL than 786-O cells. Thus, they were treated with 10 or 20 ng/mL TRAIL for 12 h after pre-treatment with 100 or 200 μg/mL GEF for the same duration. The treatment with 200 μg/mL GEF followed by a 10 and 20 ng/mL TRAIL treatment decreased the A498 cell viability to 47.55 ± 2.57% and 21.77 ± 4.68%, respectively (Figure 1B). Even after the 100 μg/mL GEF pre-treatment, the A498 cell viability significantly reduced with the subsequent TRAIL treatment (Figure 1B). Therefore, for further investigations, A498 cells were pre-treated with 200 μg/mL GEF for 12 h, followed by treatment with 10 ng/mL TRAIL for another 12 h.

The morphologies of both 786-O and A498 cells were altered after 12 h of pre-treatment with GEF, followed by a TRAIL treatment for another 12 h, whereas no significant changes were observed when treated individually (Figure 1C). Moreover, the combined TRAIL treatment following GEF pre-treatment decreased both the 786-O and A498 cell counts (Figure 1C). These findings highlight the ability of GEFs to sensitize RCC cells to TRAIL, indicating a synergistic effect in suppressing RCC cell proliferation.

### 3.3. GEF Synergistically Potentiates TRAIL-Induced Inhibition of Colony Formation and Cell Migration in Human RCC Cells

**Figure 2:**
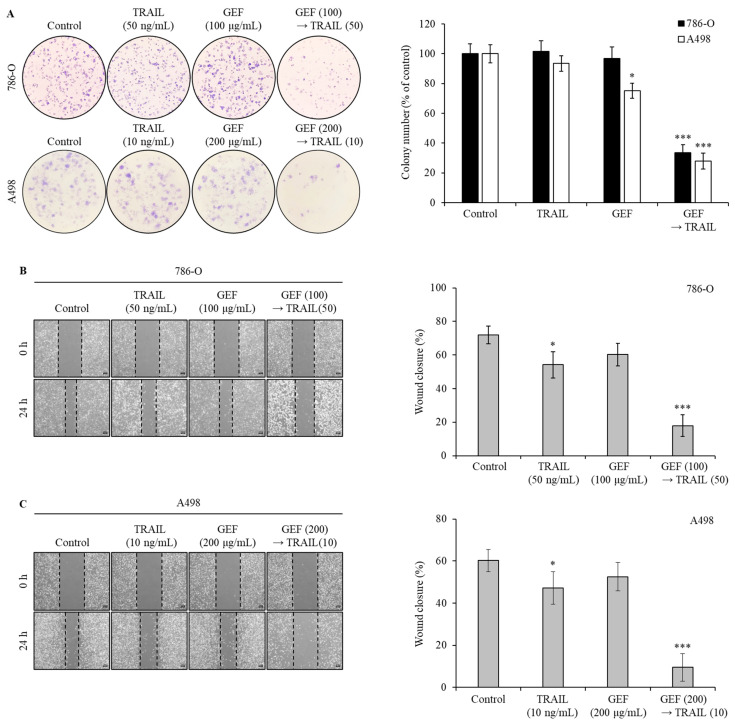
The combination of gintonin-enriched *Panax ginseng* extract fraction (GEF) and tumor necrosis factor-related apoptosis-inducing ligand (TRAIL) suppresses the clonogenic formation and migration of human renal cell carcinoma (RCC) cells. (**A**) 786-O and A498 cells were pre-treated with and without GEF for 12 h, followed by TRAIL treatment with and without GEF for an additional 12 h before reseeding. The colony formation results are shown on the left, with the percentage of stained colonies displayed in the graph on the right. (**B**,**C**) The cells were scratched and pre-treated similarly before wound size measurement. The data are represented as the mean ± standard deviation (SD) of three independent experiments, with statistical significance denoted as * *p* < 0.05, and *** *p* < 0.001 compared to the untreated control group.

To assess the effect of GEF and TRAIL on colony formation and migration in 786-O and A498 RCC cells, we performed colony formation and wound healing assays. In both assays, pre-treatment with GEF followed by TRAIL treatment markedly suppressed colony formation (Figure 2A). While individual GEF or TRAIL administration did not significantly alter colony formation, the colony count reduced to 33.54 ± 5.32% and 27.89 ± 5.33% in the 786-O and A498 cells, respectively, upon TRAIL treatment following GEF pre-treatment.

Wound healing assays showed that individual GEF or TRAIL treatments modestly reduced wound closure rates in 786-O (Figure 2B) and A498 (Figure 2C) cells compared to the controls. However, the wound closure rates significantly decreased in both cell lines following GEF pre-treatment followed by TRAIL treatment (Figure 2B,C), highlighting the potent inhibitory effects of the combination of GEF and TRAIL on cell growth and migration.

### 3.4. GEF Augments TRAIL-Induced Apoptosis and DNA Fragmentation in Human RCC Cells

**Figure 3:**
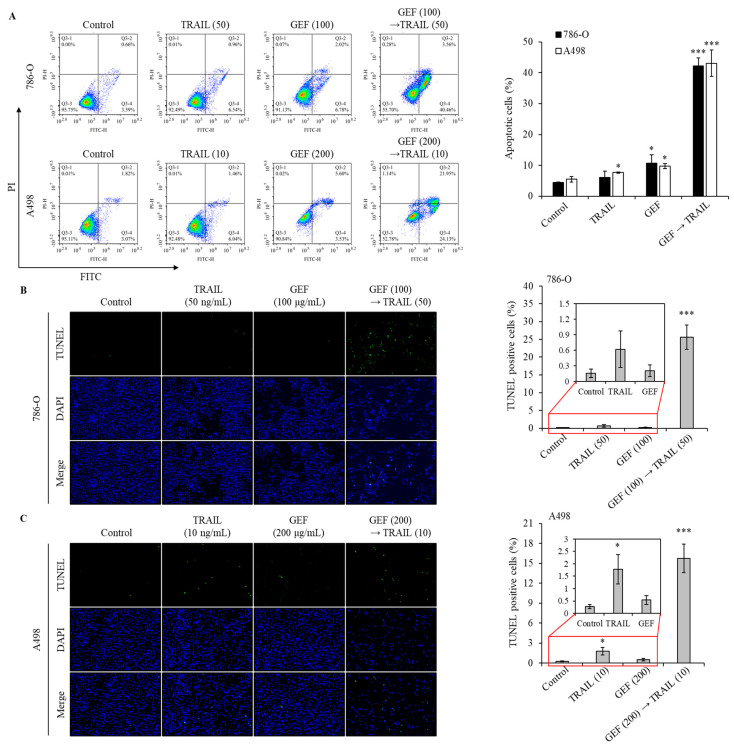
The combination of GEF and tumor necrosis factor-related apoptosis-inducing ligand (TRAIL) triggers apoptosis and DNA fragmentation in human renal cell carcinoma (RCC) cells. (**A**) 786-O and A498 cells were pre-treated with and without GEF for 12 h, followed by TRAIL treatment with and without GEF for another 12 h. Flow cytometry analysis was conducted to assess cell apoptosis. (**B**,**C**) The cells underwent similar pre-treatment before terminal deoxynucleotidyl transferase-mediated dUTP nick-end labeling (TUNEL) staining to observe DNA fragmentation. The proportion of TUNEL-positive cells was quantified. The data are represented as the mean ± standard deviation (SD) of three independent experiments, with statistical significance denoted as * *p* < 0.05, and *** *p* < 0.001 compared to the untreated control group.

Apoptosis assays using Annexin V/PI staining revealed a significant increase in the apoptotic cell population in both 786-O and A498 cells following pre-treatment with GEF and subsequent TRAIL administration (Figure 3A). The apoptotic cell population is represented by cells in the Q3-4 (early apoptosis) and Q3-2 (late apoptosis) quadrants for both cell lines. Treatment with GEF or TRAIL alone marginally increased apoptosis. However, the GEF-TRAIL combination significantly increased a proportion of apoptotic cells (early apoptosis and late apoptosis) in both cell lines. Notably, the distribution of apoptotic cell populations differed between the two cell lines: 786-O cells exhibited a higher proportion of early apoptotic cells and a lower proportion of late apoptotic cells than A498 cells, respectively.

The terminal deoxynucleotidyl transferase-mediated dUTP nick-end labeling (TUNEL) assay confirmed that GEF amplified TRAIL-induced DNA fragmentation in 786-O and A498 cells. Treatment with TRAIL following GEF pre-treatment significantly increased the number of TUNEL-positive cells in both cell lines (Figure 3B,C). Notably, TRAIL treatment alone noticeably increased the number of TUNEL-positive A498 cells, indicating sensitivity to TRAIL-induced apoptosis. These findings highlight the potential of GEF to enhance TRAIL-induced apoptosis and DNA fragmentation in RCC cells.

### 3.5. GEF Upregulates DR4/5 Expression in Human RCC Cells

**Figure 4:**
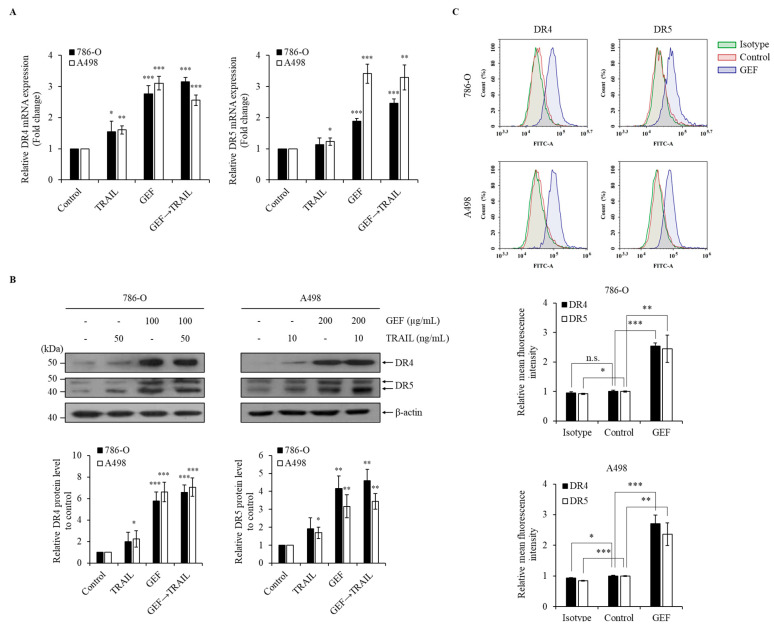
Gintonin-enriched *Panax ginseng* extract fraction (GEF) elevated death receptors 4 and 5 (DR4/5) expression in human renal cell carcinoma (RCC) cells. (**A**) 786-O and A498 cells underwent 12 h pre-treatment with GEF, followed by 12 h treatment with tumor necrosis factor-related apoptosis-inducing ligand (TRAIL). RT-qPCR was used to analyze the DR4 and DR5 mRNA levels. (**B**) Cells received pre-treatment with GEF for 12 h before exposure to various TRAIL concentrations for another 12 h. Western blot analysis quantified the DR4 and DR5 protein levels relative to that in the controls. (**C**) After a 24 h treatment with GEF or DMSO, flow cytometry assessed the cell surface expression of DR4 and DR5. The data are represented as the mean ± standard deviation (SD) of three independent experiments, with statistical significance denoted as * *p* < 0.05, ** *p* < 0.01, and *** *p* < 0.001; n.s., non-significant compared to the untreated control group.

GEF treatment significantly upregulated DR4/5 mRNA and protein levels in 786-O and A498 cells (Figure 4A,B). Furthermore, cell surface staining revealed a >two-fold increase in DR4 and DR5 expression following GEF treatment compared to that in the control groups (Figure 4C). These findings confirm that GEF enhances DR4/5 expression in RCC cells.

### 3.6. Combination of GEF and TRAIL Modulates Apoptotic Protein Expression in Human RCC Cells

**Figure 5:**
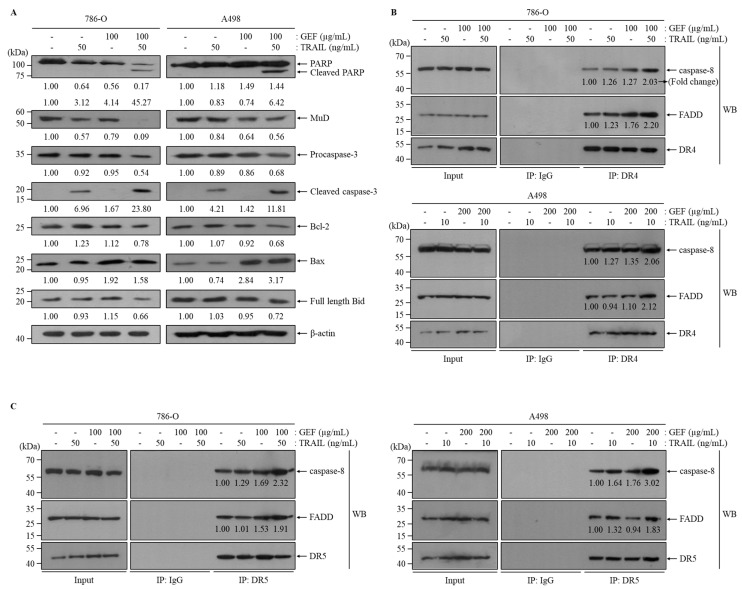
The combination of gintonin-enriched *Panax ginseng* extract fraction (GEF) and tumor necrosis factor-related apoptosis-inducing ligand (TRAIL) regulated the expression of apoptotic proteins. (**A**) 786-O and A498 cells underwent a 12 h pre-treatment with specified GEF concentrations, followed by a 12 h treatment with specified TRAIL concentrations. Western blot analysis assessed the apoptotic protein expression levels relative to the controls. (**B**,**C**) Cells received 12 h of pre-treatment with specified GEF concentrations before exposure to specified TRAIL concentrations for an additional 12 h. Immunoprecipitation using death receptors 4 (DR4) and DR5 Abs was followed by Western blot analysis of caspase-8 and Fas-associated death domain (FADD). The fold change of caspase-8 and FADD was normalized to DR4 or DR5 and to the untreated control group (=1.00).

Western blot analysis confirmed that the combination of GEF and TRAIL increased cleaved PARP, cleaved caspase-3, and Bax expression and decreased MuD, Bcl-2, and full-length Bid expression in both 786-O and A498 cells (Figure 5A). Notably, the GEF treatment alone decreased MuD expression but increased Bax expression (Figure 5A).

Immunoprecipitation assays targeting DR4 and DR5 were performed to elucidate their interactions with Fas-associated death domain (FADD) and caspase-8 in 786-O and A498 cells. GEF pre-treatment followed by TRAIL treatment significantly enhanced the interactions of FADD/caspase-8 with DR4 and DR5 (Figure 5B,C). These results suggest that the combination of GEF and TRAIL promotes apoptosis by facilitating the binding of TRAIL to DR4/5, leading to the recruitment of FADD and caspase-8, which are key components of the extrinsic apoptosis pathway in RCC cells. Overall, these findings highlight the potential of GEF and TRAIL combination therapy to induce apoptosis by modulating apoptotic proteins involved in both extrinsic and intrinsic pathways.

### 3.7. Apoptosis Induced by GEF/TRAIL in Human RCC Cells Relies on Caspase Activity

**Figure 6:**
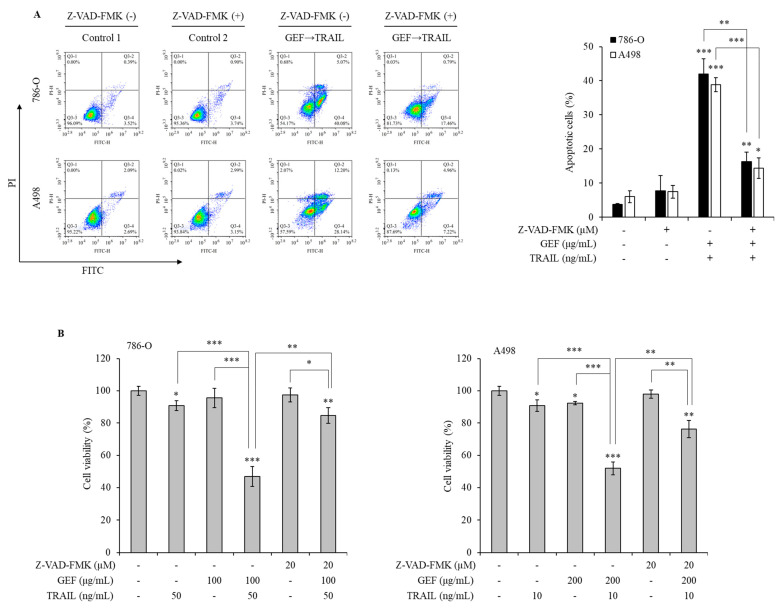
Gintonin-enriched *Panax ginseng* extract fraction (GEF)/ tumor necrosis factor-related apoptosis-inducing ligand (TRAIL)-induced apoptosis relies on the caspase activity in human renal cell carcinoma (RCC) cells. (**A**) 786-O and A498 cells were pre-treated with 20 µM Z-VAD-FMK for 1 h, followed by 12 h treatment with and without GEF. Subsequently, the cells received a 12 h treatment with and without TRAIL. Cell apoptosis was quantified using flow cytometry. (**B**) 786-O and A498 cells underwent a 1 h pre-treatment with 20 µM Z-VAD-FMK, followed by a 12 h treatment with specified GEF concentrations. A subsequent 12 h treatment with specified TRAIL concentrations was performed, and the cell viability was assessed using the WST-1 assay. The data are represented as the mean ± standard deviation (SD) of three independent experiments, with statistical significance denoted as * *p* < 0.05, ** *p* < 0.01, and *** *p* < 0.001 compared to the untreated control group.

Pan-caspase inhibitors were used to explore the mechanism of GEF/TRAIL-induced apoptosis in 786-O and A498 cells. The combination treatment significantly increased the apoptotic cell population compared to that in the controls in both cell lines. However, pre-treatment with Z-VAD-FMK, a pan-caspase inhibitor, neutralized this effect (Figure 6A), indicating the involvement of caspase activation in apoptosis.

The WST-1 assay demonstrated a significant reduction in cell viability following the GEF pre-treatment and TRAIL treatment, which was reversed by the Z-VAD-FMK pre-treatment in both cell lines (Figure 6B). These results highlight the caspase-dependent mechanism underlying GEF/TRAIL-induced apoptosis in human RCC cells.

## 4. Discussion

We have previously detailed the extraction method and characteristics of the extracted GEF [20,30]. To summarize briefly, GEF comprises approximately 7.5% linoleic acid (C18:2), 2.8% palmitic acid (C16:0), and 1.5% oleic acid (C18:1). In addition, GEF contains around 0.2% lysophosphatidic acid (LPA) C18:2, 0.06% LPA C16:0, and 0.02% LPA C18:1. It also includes 0.13% lysophosphatidylinositol, 0.08% lysophosphatidylcholine, and 0.03% lysophosphatidylethanolamine. Furthermore, GEF consists of approximately 1% phosphatidic acid (PA) 16:0-18:2, 0.5% PA 18:2-18:2, and 0.2% PA 16:0-18:1. Our research indicates that LPA C18:2 is a significant active component in gintonin. We have shown that GEF affects neuronal cells via LPA receptor activation [32] and stimulates insulin secretion through mechanisms that do not involve LPA receptors, implying the presence of other bioactive lipids in GEF besides LPAs [30].

In this study, we investigated how GEF affects TRAIL-induced apoptosis in human RCC cells. Our results demonstrate that GEF enhanced DR4/5 expression and boosted the decreased MuD expression as well as TRAIL-triggered caspase-dependent apoptosis in these cells. This highlights the potential of GEFs to sensitize RCC cells to TRAIL-induced apoptosis and offers a promising therapeutic strategy. Considering that TRAIL selectively induces apoptosis in cancer cells and the observed resistance in various cancer types, the synergistic effect of GEF and TRAIL holds significant therapeutic promise.

Ginseng-derived GEF exhibits diverse biological effects, including anti-inflammatory and anticancer properties [22,24]. Our study revealed the novel role of GEF as a sensitizer to TRAIL-induced apoptosis by upregulating DR4 and 5, which are essential for the extrinsic apoptosis pathway and TRAIL targeting [33,34,35,36]. Consistent with previous findings [33,34], GEF-induced DR4/5 upregulation enhanced TRAIL-induced apoptosis (Figure 4). Additionally, the combined GEF and TRAIL treatment not only inhibited cell proliferation, colony formation, and migration but also intensified DNA fragmentation (Figure 1, Figure 2 and Figure 3), highlighting its potent apoptotic impact. Notably, the early apoptotic 786-O cell population was larger than the early apoptotic A498 cell population (Figure 3A), indicating a delayed apoptotic signaling response, possibly due to higher TRAIL resistance in 786-O cells. Furthermore, as shown in Figure 4C, the relatively TRAIL-resistant 786-O cells exhibited a significant difference only in DR5 between the isotype and control groups, whereas the TRAIL-sensitive A498 cells showed significant differences in both DR4 and DR5 between the isotype and control groups. Along with the Western blot results in Figure 4B, this indicates that both cell lines expressed death receptors in their steady state. The involvement of caspase activity, as shown by the effects of the pan-caspase inhibitor, Z-VAD-FMK, further confirmed the caspase-dependent nature of GEF/TRAIL-induced apoptosis (Figure 6).

Our investigation revealed a decrease in the levels of the anti-apoptotic protein MuD after the GEF and TRAIL treatment (Figure 5), suggesting its potential role in facilitating apoptosis. This interplay between GEF and MuD may offer novel avenues for enhancing TRAIL-based therapies for RCC. Owing to the implications of MuD in apoptotic signaling pathways [29,37] and its increased expression in kidney cancer, understanding its regulation may reveal promising therapeutic targets for RCC [38]. GEF-mediated MuD modulation presents a mechanism through which ginseng-derived compounds influence cell fate determinants, particularly in RCC, where resistance to apoptosis is a significant challenge in treatment strategies.

Our findings extend beyond RCC and highlight the potential use of GEFs in cancer therapy. GEF has diverse pharmacological effects, including anti-inflammatory activity and cancer cell metastasis inhibition. Thus, combining GEF with TRAIL is a promising strategy for TRAIL-resistant cancers [21,22,23,24]. However, further research should aim to understand the molecular mechanisms underlying the GEF-mediated DR4/5 expression modulation and their impact on MuD function. In addition, a follow-up study is needed to determine the active ingredient in GEF involved in TRAIL-mediated apoptosis. Assessing the in vivo therapeutic efficacy and potential side effects of the GEF/TRAIL combination therapy is crucial. Exploring this approach for other TRAIL-resistant cancers is promising for transformative advancements in oncology.

Recently, cancer treatment research has expanded across various fields, including targeted chemotherapy using nanotechnology and biomaterials [39,40,41]. Furthermore, numerous studies are currently exploring the potential of natural products to enhance the effectiveness of cancer immunotherapy. For instance, research by Obaidi et al. [42,43] demonstrated that curcumin could inhibit the proliferation and cell cycle of TRAIL-resistant renal cancer cells by increasing the expression of the *let-7C* gene. Similarly, studies by Xu et al. [16] and Tewary et al. [14] found that 17β-Hydroxywithanolides (17-BHW) and physachenolide (a 17-BHW class compound) make RCC cells more susceptible to TRAIL-induced apoptosis. Building on these findings, the present study suggests that GEF may sensitize TRAIL-resistant cancer cells, indicating its potential clinical application as a standalone natural anticancer agent or in combination immunotherapies.

Our study emphasizes the importance of investigating the effect of natural compounds such as GEF in cancer research. Identifying bioactive constituents from sources such as ginseng opens new avenues for developing adjunctive cancer therapies, as incorporating natural products into conventional treatments holds promise for overcoming drug resistance and improving patient outcomes.

**Figure 7:**
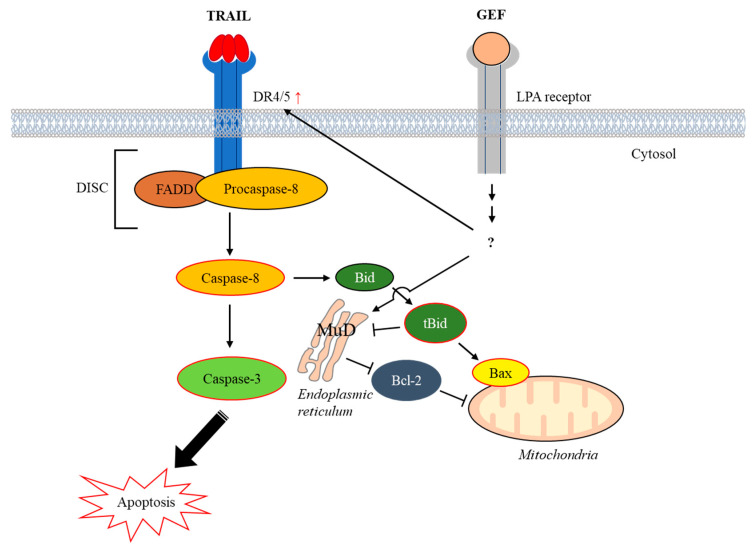
Schematic representation illustrating the induction of apoptosis by the combined action of gintonin-enriched *Panax ginseng* extract fraction (GEF) and tumor necrosis factor-related apoptosis-inducing ligand (TRAIL). Abbreviations: Bax, Bcl-2–associated X protein; Bcl-2, B-cell lymphoma-2; Bid, BH3 interacting-domain death agonist; DISC, death-inducing signaling complex; DR4/5, death receptors 4 and 5; FADD, Fas-associated death domain; LPA, lysophosphatidic acid; MuD, Mu-2-related death-inducing gene; tBid, truncated Bid.

In summary, our study demonstrates that GEF sensitizes human RCC cells to TRAIL-induced apoptosis by upregulating DR4/5 and modulating apoptotic protein expression (Figure 7). These findings suggest a promising strategy for overcoming TRAIL resistance in cancer therapy and highlight the potential of GEF as a valuable adjunct to TRAIL-based treatments.

## Figures and Tables

**Table 1:** Amounts of lipids in GEF.

Analyte	Amount (mg/g)	Content (%)
Linoleic acid	71.52 ± 2.38	7.15
Palmitic acid	26.77 ± 0.76	2.68
Oleic acid	15.37 ± 0.29	1.54
LPA C18:2	1.91 ± 5.33	0.19
LPA C16:0	0.60 ± 3.19	0.06
LPA C18:1	0.20 ± 0.39	0.02
LPC C18:2	0.78 ± 4.17	0.078
LPE C18:2	0.11 ± 8.13	0.011
LPE C16:0	0.20± 2.10	0.02
LPE C18:1	BSL	BSL
LPI C18:2	0.88 ± 3.16	0.088
LPI C16:0	0.40 ± 4.38	0.04
LPI C18:1	0.10 ± 5.63	0.010
PA 16:0-18:2	11.72 ± 0.90	1.17
PA 18:2-18:2	4.60 ± 4.90	0.46
PA 16:0-18:1	1.61 ± 4.46	0.16
PC 18:2-18:2	0.24 ± 1.25	0.024
PC 16:0-18:2	0.27 ± 1.54	0.027
PI 18:2-18:2	BSL	BSL
PI 16:0-16:0	BSL	BSL

Amount (mg/g) = the mean ± RSD (%) derived from measurements of three distinct white ginseng samples. Abbreviations: BSL, below the sensitivity limit; GEF, gintonin-enriched fraction; LPA, lysophosphatidic acid; LC-MS/MS, liquid chromatography–tandem mass spectrometry; LPC, lysophosphatidylcholine; LPE, lysophosphatidylethanolamine; LPI, lysophosphatidylinositol; MRM, multiple reaction monitoring; PA, phosphatidic acid; PC, phosphatidylcholine; PI, phosphatidylinositol; RSD, relative standard deviation. The quantities of LPE C18:2, LPI C18:2, and PI 18:2-18:2 were measured without the use of standards, and their identification and quantification were performed using MRM transitions. The LC-MS/MS conditions and calibration equations were established based on LPE C18:1, LPI C18:1, and PI 16:0-16:0.

## Data Availability

Data will be made available upon request.

## Supplementary Materials

The following supporting information can be downloaded at: https://www.mdpi.com/article/10.3390/cimb46100646/s1, Figure S1: TRAIL decreases the cell viability of human renal cell carcinoma (RCC) cells, Figure S2: Gintonin-enriched Panax ginseng extract fraction (GEF) decreases the cell viability of human renal cell carcinoma (RCC) cells.

## Abbreviations

Bax: Bcl-2–associated X protein; Bcl-2, B-cell lymphoma-2; Bid, BH3 interacting-domain death agonist; Co-IP, Co-immunoprecipitation; DISC, death-inducing signaling complex; DMSO, dimethyl sulfoxide; DR4/5, death receptors 4 and 5; FADD, Fas-associated death domain; FITC, fluorescein isothiocyanate; GEF, gintonin-enriched *Panax ginseng* extract fraction; LPA, lysophosphatidic acid; PVDF, polyvinylidene difluoride; MFI, mean fluorescence intensity; MUDENG, Mu-2-related death-inducing gene; RCC, renal cell carcinoma; TRAIL, tumor necrosis factor-related apoptosis-inducing ligand; TUNEL, terminal deoxynucleotidyl transferase-mediated dUTP nick-end labeling.

## References

[1] Moch H., Cubilla A.L., Humphrey P.A., Reuter V.E., Ulbright T.M. (2016). The 2016 WHO Classification of Tumours of the Urinary System and Male Genital Organs-Part A: Renal, Penile, and Testicular Tumours. Eur. Urol..

[2] Sung H., Ferlay J., Siegel R.L., Laversanne M., Soerjomataram I., Jemal A., Bray F. (2021). Global Cancer Statistics 2020: GLOBOCAN Estimates of Incidence and Mortality Worldwide for 36 Cancers in 185 Countries. CA Cancer J. Clin..

[3] Shinder B.M., Rhee K., Farrell D., Farber N.J., Stein M.N., Jang T.L., Singer E.A. (2017). Surgical Management of Advanced and Metastatic Renal Cell Carcinoma: A Multidisciplinary Approach. Front. Oncol..

[4] Finn R.S., Qin S., Ikeda M., Galle P.R., Ducreux M., Kim T.Y., Kudo M., Breder V., Merle P., Kaseb A.O. (2020). Atezolizumab plus Bevacizumab in Unresectable Hepatocellular Carcinoma. N. Engl. J. Med..

[5] Li P.X., Wong Y.N., Armstrong K., Haas N., Subedi P., Davis-Cerone M., Doshi J.A. (2016). Survival among patients with advanced renal cell carcinoma in the pretargeted versus targeted therapy eras. Cancer Med..

[6] Siegel R.L., Miller K.D., Wagle N.S., Jemal A. (2023). Cancer statistics, 2023. CA Cancer J. Clin..

[7] Johnstone R.W., Frew A.J., Smyth M.J. (2008). The TRAIL apoptotic pathway in cancer onset, progression and therapy. Nat. Rev. Cancer.

[8] Ashkenazi A. (2015). Targeting the extrinsic apoptotic pathway in cancer: Lessons learned and future directions. J. Clin. Investig..

[9] LeBlanc H.N., Ashkenazi A. (2003). Apo2L/TRAIL and its death and decoy receptors. Cell Death Differ..

[10] Huang Y., Sheikh M.S. (2007). TRAIL death receptors and cancer therapeutics. Toxicol. Appl. Pharmacol..

[11] Smyth M.J., Takeda K., Hayakawa Y., Peschon J.J., van den Brink M.R., Yagita H. (2003). Nature’s TRAIL--on a path to cancer immunotherapy. Immunity.

[12] Haque I., Subramanian A., Huang C.H., Godwin A.K., Van Veldhuizen P.J., Banerjee S., Banerjee S.K. (2017). The Role of Compounds Derived from Natural Supplement as Anticancer Agents in Renal Cell Carcinoma: A Review. Int. J. Mol. Sci..

[13] Feng C., Lyu Y., Gong L., Wang J. (2022). Therapeutic Potential of Natural Products in the Treatment of Renal Cell Carcinoma: A Review. Nutrients.

[14] Tewary P., Brooks A.D., Xu Y.M., Wijeratne E.M.K., Babyak A.L., Back T.C., Chari R., Evans C.N., Henrich C.J., Meyer T.J. (2021). Small-Molecule Natural Product Physachenolide C Potentiates Immunotherapy Efficacy by Targeting BET Proteins. Cancer Res..

[15] Wijeratne E.M.K., Xu Y.M., Liu M.P.X., Inacio M.C., Brooks A.D., Tewary P., Sayers T.J., Gunatilaka A.A.L. (2021). Ring A/B-Modified 17β-Hydroxywithanolide Analogues as Antiproliferative Agents for Prostate Cancer and Potentiators of Immunotherapy for Renal Carcinoma and Melanoma. J. Nat. Prod..

[16] Xu Y.M., Brooks A.D., Wijeratne E.M.K., Henrich C.J., Tewary P., Sayers T.J., Gunatilaka A.A.L. (2017). 17β-Hydroxywithanolides as Sensitizers of Renal Carcinoma Cells to Tumor Necrosis Factor-α Related Apoptosis Inducing Ligand (TRAIL) Mediated Apoptosis: Structure-Activity Relationships. J. Med. Chem..

[17] Zhang H., Abid S., Ahn J.C., Mathiyalagan R., Kim Y.J., Yang D.C., Wang Y. (2020). Characteristics of Panax ginseng Cultivars in Korea and China. Molecules.

[18] Nah S.Y. (2012). Gintonin: A novel ginseng-derived ligand that targets G protein- coupled lysophosphatidic acid receptors. Curr. Drug Targets.

[19] Nah S.Y., Kim D.H., Rhim H. (2007). Ginsenosides: Are any of them candidates for drugs acting on the central nervous system?. CNS Drug Rev..

[20] Choi S.H., Jung S.W., Kim H.S., Kim H.J., Lee B.H., Kim J.Y., Kim J.H., Hwang S.H., Rhim H., Kim H.C. (2015). A brief method for preparation of gintonin-enriched fraction from ginseng. J. Ginseng Res..

[21] Kim H.J., Choi S.H., Lee N.E., Cho H.J., Rhim H., Kim H.C., Hwang S.H., Nah S.Y. (2020). Effects of Gintonin-Enriched Fraction on Methylmercury-Induced Neurotoxicity and Organ Methylmercury Elimination. Int. J. Environ. Res. Public Health.

[22] Chei S., Song J.H., Oh H.J., Lee K., Jin H., Choi S.H., Nah S.Y., Lee B.Y. (2020). Gintonin-Enriched Fraction Suppresses Heat Stress-Induced Inflammation through LPA Receptor. Molecules.

[23] Kim H., Lee B.H., Choi S.H., Kim H.J., Jung S.W., Hwang S.H., Rhim H., Kim H.C., Cho I.H., Nah S.Y. (2015). Gintonin stimulates gliotransmitter release in cortical primary astrocytes. Neurosci. Lett..

[24] Hwang S.H., Lee B.H., Kim H.J., Cho H.J., Shin H.C., Im K.S., Choi S.H., Shin T.J., Lee S.M., Nam S.W. (2013). Suppression of metastasis of intravenously-inoculated B16/F10 melanoma cells by the novel ginseng-derived ingredient, gintonin: Involvement of autotaxin inhibition. Int. J. Oncol..

[25] Hirst J., Barlow L.D., Francisco G.C., Sahlender D.A., Seaman M.N.J., Dacks J.B., Robinson M.S. (2011). The Fifth Adaptor Protein Complex. PLoS Biol..

[26] Kawasaki H., Taira K. (2002). A functional gene discovery in the Fas-mediated pathway to apoptosis by analysis of transiently expressed randomized hybrid-ribozyme libraries. Nucleic Acids Res..

[27] Lee M.R., Shin J.N., Moon A.R., Park S.Y., Hong G., Lee M.J., Yun C.W., Seol D.W., Piya S., Bae J. (2008). A novel protein, MUDENG, induces cell death in cytotoxic T cells. Biochem. Biophys. Res. Commun..

[28] Shin J.N., Han J.H., Kim J.Y., Moon A.R., Kim J.E., Chang J.H., Bae J., Oh J.W., Kim T.H. (2013). MUDENG is cleaved by caspase-3 during TRAIL-induced cell death. Biochem. Biophys. Res. Commun..

[29] Choi J.H., Lim J.B., Wickramanayake D.D., Wagley Y., Kim J., Lee H.C., Seo H.G., Kim T.H., Oh J.W. (2016). Characterization of MUDENG, a novel anti-apoptotic protein. Oncogenesis.

[30] Cho H.J., Choi S.H., Kim H.J., Lee B.H., Rhim H., Kim H.C., Hwang S.H., Nah S.Y. (2019). Bioactive lipids in gintonin-enriched fraction from ginseng. J. Ginseng Res..

[31] Wagley Y., Choi J.H., Wickramanayake D.D., Choi G.Y., Kim C.K., Kim T.H., Oh J.W. (2013). A monoclonal antibody against human MUDENG protein. Monoclon. Antib. Immunodiagn. Immunother..

[32] Choi S.H., Jung S.W., Lee B.H., Kim H.J., Hwang S.H., Kim H.K., Nah S.Y. (2015). Ginseng pharmacology: A new paradigm based on gintonin-lysophosphatidic acid receptor interactions. Front. Pharmacol..

[33] Deng Y., Bi R., Guo H., Yang J., Du Y., Wang C., Wei W. (2019). Andrographolide Enhances TRAIL-Induced Apoptosis via p53-Mediated Death Receptors Up-Regulation and Suppression of the NF-small ka, CyrillicB Pathway in Bladder Cancer Cells. Int. J. Biol. Sci..

[34] Kim B.R., Park S.H., Jeong Y.A., Na Y.J., Kim J.L., Jo M.J., Jeong S., Yun H.K., Oh S.C., Lee D.H. (2019). RUNX3 enhances TRAIL-induced apoptosis by upregulating DR5 in colorectal cancer. Oncogene.

[35] Ren Y., Wang X., Huang S., Xu Y., Weng G., Yu R. (2021). Alternol Sensitizes Renal Carcinoma Cells to TRAIL-Induced Apoptosis. Front. Pharmacol..

[36] Kim B., Seo J.H., Lee K.Y., Park B. (2020). Icariin sensitizes human colon cancer cells to TRAIL-induced apoptosis via ERK-mediated upregulation of death receptors. Int. J. Oncol..

[37] Muthu M., Chun S., Gopal J., Park G.S., Nile A., Shin J., Shin J., Kim T.H., Oh J.W. (2020). The MUDENG Augmentation: A Genesis in Anti-Cancer Therapy?. Int. J. Mol. Sci..

[38] Lv Y., Lin S.Y., Hu F.F., Ye Z., Zhang Q., Wang Y., Guo A.Y. (2020). Landscape of cancer diagnostic biomarkers from specifically expressed genes. Brief. Bioinform..

[39] Venkatesan P., Thirumalaivasan N., Yu H.P., Lai P.S., Wu S.P. (2019). Redox Stimuli Delivery Vehicle Based on Transferrin-Capped MSNPs for Targeted Drug Delivery in Cancer Therapy. ACS Appl. Bio Mater..

[40] Thirumalaivasan N., Venkatesan P., Lai P.S., Wu S.P. (2019). In Vitro and In Vivo Approach of Hydrogen-Sulfide-Responsive Drug Release Driven by Azide-Functionalized Mesoporous Silica Nanoparticles. ACS Appl. Bio Mater..

[41] Murugesan V., Govindarasu M., Manoharadas S., Pandiaraj S., Thiruvengadam M., Govindasamy R., Vaiyapuri M. (2023). Combinatorial Anticancer Effects of Multi Metal Ion and Drug Substitute with Hydroxyapatite Coatings on Surgical Grade 316LSS Stainless Steel Alloys towards Biomedical Applications. J. Mater. Res. Technol..

[42] Obaidi I., Cassidy H., Gaspar V.I., McCaul J., Higgins M., Halasz M., Reynolds A.L., Kennedy B.N., McMorrow T. (2020). Curcumin Sensitizes Kidney Cancer Cells to TRAIL-Induced Apoptosis via ROS Mediated Activation of JNK-CHOP Pathway and Upregulation of DR4. Biology.

[43] Obaidi I., Blanco Fernandez A., McMorrow T. (2022). Curcumin Sensitises Cancerous Kidney Cells to TRAIL Induced Apoptosis via Let-7C Mediated Deregulation of Cell Cycle Proteins and Cellular Metabolism. Int. J. Mol. Sci..

