# Supplementary material for: Gintonin-Enriched Panax ginseng Extract Fraction Sensitizes Renal Carcinoma Cells to TRAIL-Induced Apoptosis through DR4/5 Upregulation
Source: Curr Issues Mol Biol. 2024 Sep 27;46(10):10880–95. doi: 10.3390/cimb46100646 (PMC11506827; doi:10.3390/cimb46100646)
Supplement: Supplementary file 1 [file cimb-46-00646-s001.zip › cimb-3227315-supplementary.pdf]

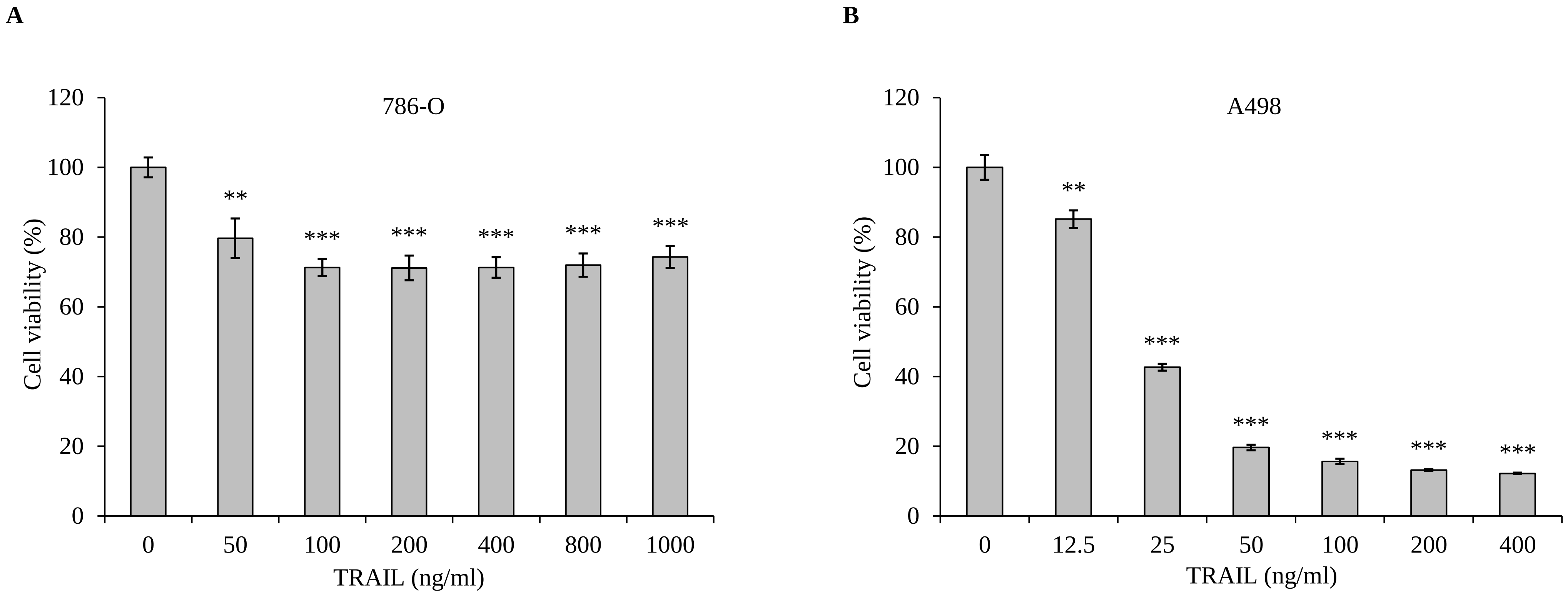

Supplementary Figure S1. TRAIL decreases the cell viability of human renal cell carcinoma (RCC) cells. (**a, b**) 786-O and A498 cells were treated with varying TRAIL concentrations for 24 h. Cell viability was assessed using the WST-1 assay. Data represent mean ± standard deviation (SD) of three independent experiments, with statistical significance denoted as **$P<0.01$, ***$P<0.001$ compared to the untreated control group.

A

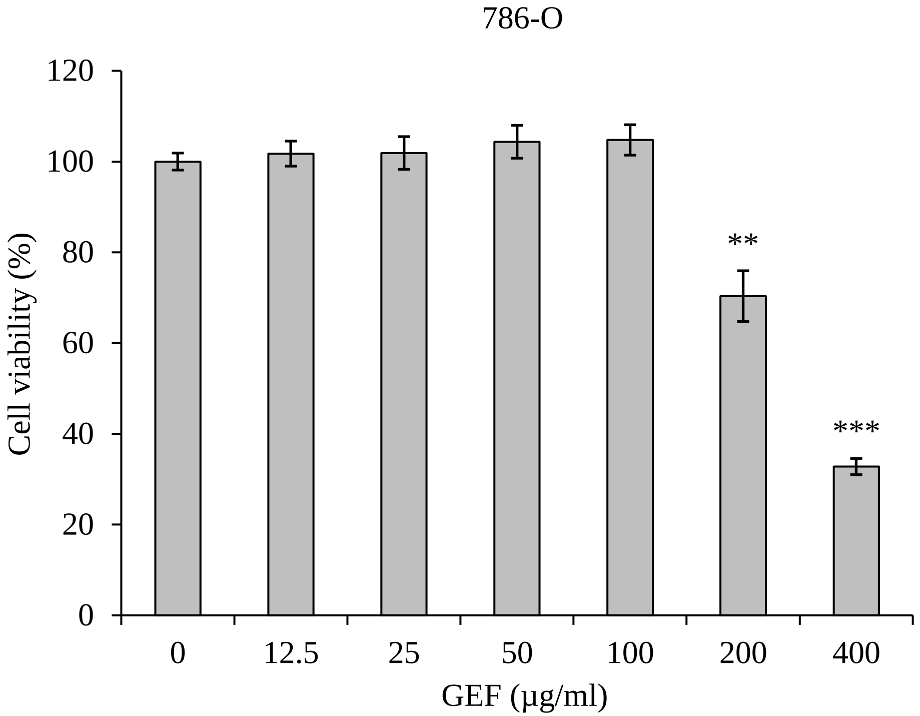

B

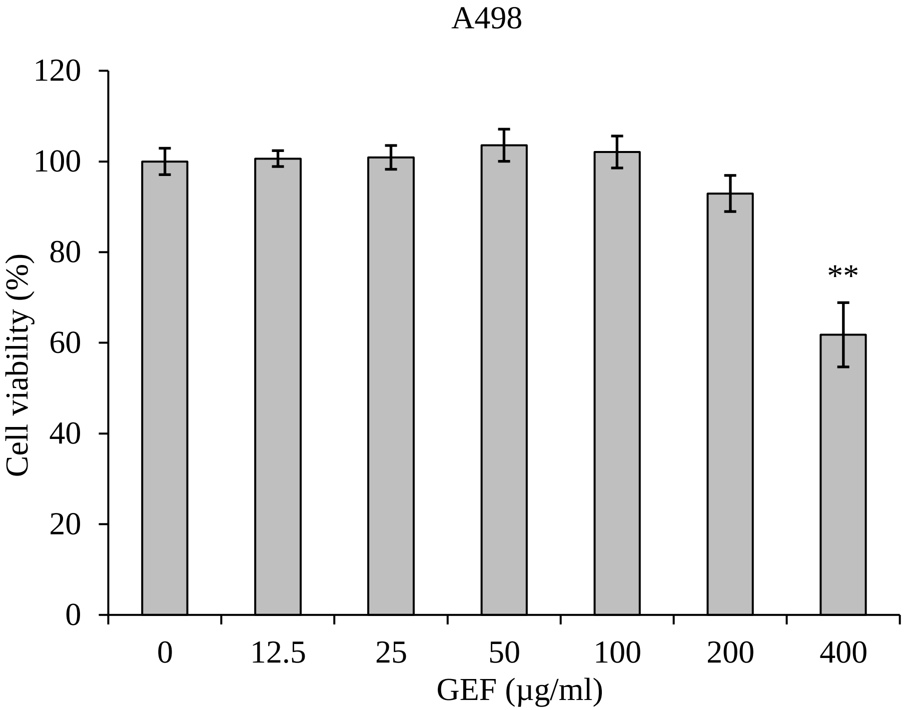

Supplementary Figure S2. Gintonin-enriched Panax ginseng extract fraction **(**GEF) decreases the cell viability of human renal cell carcinoma (RCC) cells. (**a, b**) 786-O and A498 cells were treated with varying GEF concentrations for 24 h. Cell viability was assessed using the WST-1 assay. Data represent mean ± standard deviation (SD) of three independent experiments, with statistical significance denoted as **P<0.01, ***P<0.001 compared to the untreated control group.
